# Non-Coding RNAs and Their Role in Respiratory Syncytial Virus (RSV) and Human Metapneumovirus (hMPV) Infections

**DOI:** 10.3390/v12030345

**Published:** 2020-03-21

**Authors:** Wenzhe Wu, Eun-Jin Choi, Inhan Lee, Yong Sun Lee, Xiaoyong Bao

**Affiliations:** 1 Department of Pediatrics, The University of Texas Medical Branch, Galveston, TX 77555, USA; wenwu@utmb.edu (W.W.); euchoi@utmb.edu (E.-J.C.); 2 miRcore, Ann Arbor, MI 48105, USA; inhanlee99@gmail.com; 3 Department of Cancer System Science, Graduate School of Cancer Science and Policy, National Cancer Center, Goyang-si Gyeonggi-do 10408, Korea; yslee@ncc.re.kr; 4 Sealy Center for Molecular Medicine, The University of Texas Medical Branch, Galveston, TX 77555, USA; 5 The Institute of Translational Sciences, The University of Texas Medical Branch, Galveston, TX 77555, USA; 6 The Institute for Human Infections and Immunity, The University of Texas Medical Branch, Galveston, TX 77555, USA

**Keywords:** RSV, hMPV, ncRNAs

## Abstract

Recent high-throughput sequencing revealed that only 2% of the transcribed human genome codes for proteins, while the majority of transcriptional products are non-coding RNAs (ncRNAs). Herein, we review the current knowledge regarding ncRNAs, both host- and virus-derived, and their role in respiratory syncytial virus (RSV) and human metapneumovirus (hMPV) infections. RSV is known as the most common cause of lower respiratory tract infection (LRTI) in children, while hMPV is also a significant contributor to LRTI in the pediatrics population. Although RSV and hMPV are close members, belonging to the *Pneumoviridae* family, they induce distinct changes in the ncRNA profile. Several types of host ncRNAs, including long ncRNA (lncRNA), microRNAs (miRNAs), and transfer RNA (tRNA)-derived RNA fragments (tRFs), are involved as playing roles in RSV and/or hMPV infection. Given the importance of ncRNAs in regulating the expression and functions of genes and proteins, comprehensively understanding the roles of ncRNAs in RSV/hMPV infection could shed light upon the disease mechanisms of RSV and hMPV, potentially providing insights into the development of prevention strategies and antiviral therapy. The presence of viral-derived RNAs and the potential of using ncRNAs as diagnostic biomarkers are also discussed in this review.

## 1. Introduction

Respiratory syncytial virus (RSV) is the most common viral pathogen causing lower respiratory tract infection (LRTI) in young children, the elderly, and immune-compromised patients. Globally, RSV is estimated to cause 24.8 million cases and about 70–80 K LRTI deaths annually, and 54% of these deaths occur in children younger than 5 years [1]. Of the pathogens responsible for LRTI, RSV infection accounts for 85% of bronchiolitis and 20% of pneumonia, and is the major reason for hospitalization in infants [2]. Severe RSV infection is also associated with increased incidences of persistent wheezing, decreased lung function, asthma, and possibly allergic sensitization later in life [3,4,5,6,7]. To date, there are no effective vaccines or specific drugs against RSV. Palivizumab, a humanized monoclonal antibody, is the only Food and Drug Administration (FDA)-approved measure for preventing severe RSV-associated respiratory illness. However, RSV prophylaxis with palivizumab is only recommended for infants that are at high risk because of prematurity or other medical problems, such as congenital heart disease [8]. Human metapneumovirus (hMPV) is another major cause of bronchiolitis in early childhood. In some reports, it is claimed to be second only to RSV [9]. hMPV is responsible for ~10% of LRTI requiring hospitalization in children [10]. Seroepidemiology studies indicate that >90% of children have been infected with hMPV by 5 years of age [11]. Clinical studies indicate that hMPV infection is also linked to wheezing and asthma exacerbations in children and adults [12,13,14,15]. Other than the pediatrics population, patients with immunologic disorders, defects of the heart and/or lung structures, and organ transplantation are also high-risk groups to develop severe diseases following RSV or hMPV infection [16,17]. Currently, there are no licensed vaccines or antiviral therapies for hMPV, and the treatment is only supportive.

There are many differences between the RSV and hMPV, although their clinical manifestations, including rhinorrhea, cough, fever, and respiratory distress, are often indistinguishable in patients [18]. For example, the peak age of hMPV-infected children is about 13–24 months old, while the peak age of RSV is 0–3 months old [19]. The incidence peak of RSV is in winter, while hMPV peaks in spring [16]. The blood eosinophil level in RSV patients is higher than that in hMPV patients [20]. Moreover, RSV and hMPV trigger different host immune responses [21,22,23,24].

The majority (98%) of the mammalian genome is transcribed to RNAs without coding potential, known as non-coding RNAs (ncRNAs) [25]. ncRNAs can function as regulators of translation, RNA splicing, and gene expression by participating in transcriptional and post-transcriptional gene regulation, heterochromatin formation, histone modification, and DNA methylation [26,27,28]. In the last two decades, extensive studies have provided numerous evidence for the involvement of ncRNAs in virtually all biological pathways [29,30], including proliferation, differentiation, apoptosis, autophagy, tissue repair and remodeling, and immune responses.

According to the length, ncRNAs could be classified into small ncRNAs (sncRNAs, <200 nt) and long ncRNAs (lncRNAs, > 200 nt). MicroRNAs (miRNAs), Piwi-interacting RNAs (piRNAs), small nucleolar RNAs (snoRNAs), and recently discovered tRNA-derived RNA fragments (tRFs) all belong to sncRNAs. The infection of RSV or hMPV can significantly alter the expression profile of host ncRNAs and some impacted ncRNAs have been shown to play significant roles in viral replication and/or host responses. In this review, we provide an overview of the latest knowledge and summarize existing data on ncRNAs involved in RSV and hMPV infections.

## 2. miRNAs in RSV and hMPV Infections

miRNAs, the most widely studied sncRNAs, are endogenous, single-stranded ncRNAs of 18–24 nt in length. They are usually guided by Dicer to be loaded into the Argonaute protein complex termed RNA-induced silencing complex (RISC) to carry out their function [31]. The loaded miRNAs then use their “seed sequence”, a conserved six-eight base sequence which is mostly situated at the 5′-end of the miRNA, to recognize and bind to special sequences within the 3′ or 5′ untranslated or coding region of messenger RNA (mRNA) targets via partial complementary or complementary base-pairing [32]. miRNA/RISC-mRNA interactions lead to post-transcriptional gene silencing, including mRNA degradation/cleavage or preventing mRNA from being translated [33]. Single miRNAs can potentially target over 300 different transcripts [34]. Additionally, novel and unconventional functions of miRNAs keep emerging, such as epigenetic modification, the promotion of efficient splicing of nascent mRNA, and their regulation on the expression/functions of other ncRNAs [35]. Numerous pieces of evidence demonstrate that miRNAs impinge on nearly all biological processes in mammals. Indeed, approximately 60% of protein-coding genes in the human genome are more or less controlled by miRNAs, and the altered expression of miRNAs has been observed in various diseases, such as cancer, cardiac pathologies, airway diseases, and viral infections [36,37,38,39].

### 2.1. Both RSV and hMPV Alter Global miRNAs Expression Profile

Both microarrays and high-throughput sequencing techniques have been used to identify the changes in miRNA expression in response to RSV/hMPV infection. Altered miRNAs by RSV and hMPV are listed in Tables S1 and S2, respectively. Changes of miRNA expression by RSV infection have been demonstrated in a variety of cell types, including cell lines, such as A549, Hep2, and polarized Calu-3 cells, and primary cultured cells, such as normal human bronchial epithelial cells (NHBEs), small alveolar epithelial cells (SAECs), and monocyte-derived dendritic cells (moDCs) from normal human peripheral blood mononuclear cells (PBMCs) [40,41,42,43,44,45,46]. Clinical patient samples, including those from nasal mucosa, peripheral blood, and PBMCs specimens, were also used to study the miRNA changes in response to RSV infection [47,48,49,50].

Changes of miRNA in airway epithelial cells (AECs) by RSV. AECs are the primary targets of RSV [51]. They sense the infection through pattern-recognition receptors (PRRs), including cytosolic RIG-I-like receptors and Toll-like receptors (TLRs) on the surface of cells, and then activate innate host responses, which can induce a cascade of chemotactic factors for the recruitment of a series of innate immune cells to infected sites [52,53,54]. Therefore, studying the changes in miRNAs and the function of miRNAs in ACEs is significantly important in understudying the disease mechanisms of RSV.

The impact of RSV on miRNA expression was first investigated in a miRNA microarray platform. Bakre et al. used a miRNA microarray to compare the miRNAs expression in mock- and RSV-infected A549 cells [42]. A549 cells are a commonly accepted cell model for respiratory virus infections. They found nine miRNAs with increased expression (fold≥ 1.5) and four miRNAs with decreased expression (fold≥ 1.5). qPCR confirmed the increase of let-7f, miR-24, miR-337-3p, miR-26b and miR-520a and the suppression of miR-198 and miR-595 by RSV. Several other studies using qPCR also revealed that RSV induces miR-24, miR-29a, and miR-6087 in A549 cells [43,44,55]. The changes in miRNA expression were also studied in Hep2 cells by Eilam-Frenkel et al., who discovered miR-146a-5p to be up-regulated by prolonged RSV infection. Compared with control Hep2 cells, let-7c, miR-345-5p, and miR-221 are downregulated by prolonged RSV infection [46].

Changes in the miRNA expression by RSV were also investigated in NHBEs by a multiplex qPCR array. 24 miRNAs including miR-221 are downregulated and 2 miRNAs are significantly upregulated by RSV [41]. These results are consistent with the discovery from Thornburg et al. who found altered miRNAs in RSV-infected NHBEs by microarray and qPCR [40]. Thornburg et al. also revealed that RSV modifies miRNA expression in a cell-type-specific manner and the induction of some miRNAs, such as let-7i and miR-30b, by RSV is dose-, time-, and replication-dependent [40].

miRNAs in AEC exosomes. Exosomes, nanovesicles derived from endosomes, are important for mediating proximal and distal cell-to-cell communication via the horizontal transfer of bioactive cargos, affecting the gene expression, metabolism and cellular functions of recipient cells [56]. Exosomes are involved in viral transmission and modulation of immune responses [57,58]. miRNAs can also be packaged into exosomes, and then be delivered to target cells [59]. Using next-generation sequencing, Chahar et al. characterized exosomes released from RSV-infected A549 and found that RSV induces significant changes in exosomal RNA composition [55]. There is a significant miRNA increase in exosomes derived from RSV-infected cells. miRNAs account for ~1.5% of total sncRNA reads in mock exosomes, whereas the miRNA percentage increases to ~14.3% in RSV exosomes [55]. By analyzing the miRNAs with >10 reads in exosomes derived from mock or RSV-infected cells, 66 miRNAs are commonly present in mock and RSV exosomes. Among these 66 miRNAs, 56 and 10 miRNAs are significantly upregulated and downregulated respectively by RSV. There are also 25 miRNAs, which are only detectable in RSV exosomes, and 9 miRNAs, which are only present in mock exosomes. The potential targets of altered miRNAs were subjected to Gene Ontology (GO) functional classification analysis, and the most significant target groups are related to DNA binding, transcriptional and post-transcriptional regulation, alternative splicing, and chromatin modification [55].

In summary, the AEC data demonstrated that RSV infection dramatically alters the expression profile of miRNA in AEC. Some RSV-altered miRNAs are common among cells. For example, let-7f is commonly induced by RSV in A549, polarized Calu-3, and SAECs [42,45,55]. miR-221 is decreased in RSV-infected NHBEs and Hep2 cells [41,46]. Some changes in miRNA seem to be time-dependent. For example, let-7c is elevated at 48 h post-infection in NHBEs [40], but is suppressed in Hep2 cells with prolonged RSV infection [46]. The mechanism underlying the time-dependent miRNA regulation by RSV is largely unknown.

RSV-changed miRNAs in patient samples. miRNAs are emerging as potential biomarkers and prognosis factors for diseases [60]. An investigation of the miRNA profile in RSV patients was recently carried out, to facilitate the discovery of novel biomarkers for RSV infection and to better understand the interplay between RSV and host responses. Inchley et al. profiled miRNAs expression in nasopharyngeal aspirate (NPA) samples from infants less than 12 months of age with acute RSV disease in Akershus, Norway [48]. Several cell types are present in NPA samples: granulocytes, squamous epithelial cells, and ciliary epithelial cells. Fourteen samples from severe RSV patients, thirteen mild RSV samples, and another thirteen samples from healthy clinical controls were analyzed by a miRNA microarray. Compared with the control group, there are eight upregulated and three downregulated miRNAs in RSV samples with mild or severe diseases. There is one miRNA, which is upregulated only by severe RSV infection but downregulated in mild disease samples. Fourteen miRNAs are significantly downregulated only in the mild RSV group (experimentally confirmed ones are listed in the Table S1). Notably, some RSV-regulated miRNAs, such as miR-203a, miR-27b, miR-29c, miR-34b, and miR-34c, are also impacted by asthma in adults [61,62]. Whether this serves as a mechanism contributing to the association of early-life RSV infection with later development of asthma needs to be investigated. Another interesting observation in this study is that miR-125 is downregulated in mild and moderate RSV disease groups, but not in the severe group. The downregulation of miR-125 may protect mild or moderate disease patients from an excessive innate immune response with a more severe disease phenotype, as miR-125a can function as a positive regulator of NF-κB and macrophage activation [63,64].

A study describing miRNAs fingerprint in the whole blood of RSV patients was done by a miRNA microarray in 2017, Age-matched healthy infants were recruited to form a control group [47]. The qPCR validated that miR-106b-5p, miR-20b-5p, and miR-342-3p are upregulated, while miR-320e, miR-320d, miR-877-5p, miR-122-5p, and miR-92b-5p are downregulated by RSV. Bioinformatics analysis also demonstrated that genes, potentially targeted by RSV-affected miRNAs, are enriched in a large number of pathways associated with inflammatory and immune processes, such as insulin signaling, TGF-β signaling, Wnt signaling, T and B cell receptor signaling, and Fc epsilon RI signaling pathways.

In another study, Zhang et al. used qPCR to study RSV-regulated miRNAs using RNA samples from the peripheral blood and NPA. The samples were collected from bronchiolitis children with RSV infection (6.8 ± 3.9 years, *n* = 104; 45% male) or healthy controls (6.5 ± 4.1 years, *n* = 40; 55% male) [49]. miR-140-5p is downregulated in both NPAs and peripheral blood samples of RSV patients and the downregulation of miR-140-5p appears to correlate with the severity of RSV disease.

miRNAs expression in PBMC samples of RSV patients were also recently explored. Liu et al. collected PBMCs from 20 bronchiolitis children infected with RSV and 20 healthy children. The group found that miR-26b is significantly induced in RSV patient samples [50]. This result is consistent with the finding of miR-26b in RSV-infected A549 [42]. In clinical NPA samples, microarray results demonstrated miR-26b to be significantly enhanced in severe RSV group. However, the qPCR validation failed [48]. Despite qPCR results, these independent studies highlighted the importance of miR-26 in RSV infection.

Summary of RSV-regulated miRNA. A miRNA family is a group of miRNAs that have a close sequence or common structure configuration. Normally, members from the same miRNA family have similar physiological functions. Many independent studies demonstrate that RSV infection induces the changes in miRNA members belonging to the let-7, miR-30, and miR-320 families. For example, let-7 family members are upregulated by RSV in clinical samples. Let-7d is enhanced in NPA samples of RSV patients [48]. Let-7f is induced by RSV in A549 cells and Calu-3 cells [42,45,55]. let-7c and let-7i are enhanced by RSV infection in NHBEs [40]. Let-7b is greatly more in RSV infected moDCs than uninfected cells [40]. In exosomes from RSV-infected A549 cells and SAECs, let-7a, let-7e, let-7f, and let-7i are also significantly higher than control cells [55].

Similar to the effect of RSV on the expression of the let-7 family, miRNAs of the miR-30 family are also commonly impacted by RSV infection. miR-30a, -30b, and -30c are significantly and respectively enhanced by RSV in normal NHBEs, moDCs, and A549 cells-derived exosomes [40,55,65]. The expression of three members of the miR-320 family (miR-320a, miR-320b, and miR-320c) is increased by RSV in A549 and exosomes derived from A549 [55], whereas the other two miRNAs of this family (miR-320d and miR-320e) are decreased in peripheral blood of RSV patients [47]. These findings suggest the key miRNA families in RSV infection and their potential as diagnostic markers and therapeutic targets.

miRNAs and their changes in AEC by hMPV infection. We recently discovered that hMPV-controlled miRNA expression is also cell-type-specific. In hMPV-infected A549, 201 upregulated miRNAs (by ≥1.5-fold) and 72 downregulated miRNAs (by ≤0.7-fold) were revealed by an ultra-high-throughput sequencing study [66]. The qPCR assays validated the induction of let-7f and miR-452 and the downregulation of miR-374a* and miR-192. We also found that the M2-2 protein of hMPV plays a significant role in the expression of miR-30a and miR-16. Although wild type hMPV(hMPV-WT) infection does not affect miR-30a and miR-16 expression, the virus lacking the M2-2 gene (hMPV-ΔM2-2) significantly increases miR-16 and miR-30a and the overexpression of M2-2 in hMPV-ΔM2-2-infected cells reverses the increase of miR-16 and miR-30a [66]. Further experiments indicated that the induction of miR-16 depends on type I IFN signaling, as the inhibition of M2-2 on miR-16 induction is impaired in U4A cells, a cell line lacking IFN signaling because of JAK-1 deficiency [66]. Comparable miR-16 expression in WT- and ΔM2-2-infected U4A cells also suggested that IRF-3 and NF-κB are not important for miR-16 induction, although M2-2 deletion resulted in more activated IRF-3 and NF-κB in U4A cells [67]. In contrast to miR-16, M2-2-controlled miR-30a expression seems to be IFN signaling independent. In the absence of JAK-1, hMPV-ΔM2-2 still induces more miR-30a than the WT virus in U4A cells.

Changes of miRNAs in immune cells by hMPV. The induction of miR-182-5p and miR-4634 by hMPV in human moDCs cells was reported recently [65]. Moreover, the miRNA profile of RSV-infected moDCs was also compared with that of hMPV-infected moDCs cells [65]. The induction of some miRNAs seems virus-specific. For example, RSV infection induces miR-30a, miR-4448, and miR-4634, without impacting miR-182-5p expression, while miR-4448 or miR-30a are not influenced by hMPV infection. The distinguished miRNAs patterns suggest that RSV and hMPV may use these miRNAs to develop virus-specific strategies to regulate cellular responses. In addition to virus-specific induction of miRNAs, some miRNAs are commonly inducible by both viruses. Among those, the predominant miRNA induced by both viruses is miR-4634.

### 2.2. Antiviral and Host Responses Controlled by miRNAs

Many RSV- and hMPV-regulated miRNAs are critical for the host responses to viral infections. For example, miR-140-5p, whose expression in NPA and peripheral blood samples are much lower in RSV patients, is critical for the regulation of pro-inflammatory responses in NHBEs because inhibiting miR-140-5p enhances pro-inflammatory responses [49]. The cells transfected with miR-140-5p inhibitor produce more TNF-α, IL-1β, IL-6, and IL-8 compared with those transfected with negative control miRNAs. Luciferase-UTR assays confirmed that TLR4, a sensor for RSV to initialize antiviral cascade, is the target gene of miR-140-5p, suggesting that the host uses downregulated miR-140-5p to enhance TLR4 expression and its antiviral signaling. miR-140-5p is also found to be inducible by IFN- α treatment [49], likely contributing to the prevention of RSV-induced IFN storm.

Let-7f, an hMPV-induced miRNA in A549 cells, is functionally important for viral replication control. Let-7f inhibitor significantly enhances hMPV replication and progeny virus production, while let-7f overexpression leads to an opposite effect [66].

Neurotrophic factors and their receptors contribute significantly to the pathophysiology of airway inflammation and hyperreactivity in response to RSV infection [68,69]. RSV infection has been shown to upregulate nerve growth factor (NGF) and its cognate high-affinity receptor tropomyosin-related kinase A (TrKA) and NGF-TrKA axis contributes to prolonged RSV infection and associated pathogenesis [70,71]. Othumpangat et al. found that RSV suppresses miR-221 to enhance the expression of NGF and TrKA, both at mRNA and protein levels. The overexpression miR-221, compared with negative control miRNA, prevents the increase in NGF and apoptosis in cells. In addition, the numbers of RSV-infected cells and progeny viruses are reduced by miR-221 overexpression [41], demonstrating the role of miRNAs in controlling the RSV-associated pathophysiology. Other miRNAs contributing to the pathogenesis mechanisms of RSV include miR-146a-5p and miR-345-5p, which are induced and suppressed by RSV infection, respectively [46]. The mechanism underlying the pathogenesis effect of miR-146a-5p is not clear. However, RSV-suppressed miR-345-5p might increase the p21 protein level to promote cell cycle arrest and prolong the RSV infection [46].

The role of RSV G protein in miRNA induction and associated host responses. In contract to the antiviral role of let-7f in hMPV infection, let-7f favors RSV infection [42]. The protein G of RSV seems important in the let-7f induction, as A549 cells infected by recombinant RSV lacking the G gene (RSV-ΔG) have less let-7f than those infected by wild type RSV. In addition, purified RSV G protein remarkably triggers the induction of let-7f in A549 [42]. These results suggest that RSV G induces more let-7f to promote RSV replication. Let-7f has been reported to directly target genes associated with cell proliferation, survival, and immune cell recruitment, such as cyclin D1 (CCND1), dual-specificity tyrosine phosphorylation regulated kinase 2 (DYRK2), E74-like factor 4 (ELF4) C-C motif chemokine ligand 7 (CCL7), and suppressor of cytokine signaling 3 (SOCS3) [72,73,74]. The deregulation of CCND1, DYRK2, and ELF4 may result in aberrant cell cycle progression, leading to RSV-induced G1 arrest [75]. In addition, ELF4 is critical for host antiviral response, which can be induced by IFN-β and upregulates IFN-β expression in a feed-forward loop [76]. The chemoattractant CCL7 is important for the recruitment of monocyte-derived cells to inflamed lung early after RSV infection [77]. SOCS proteins have been identified as inducible feedback inhibitors of cytokine receptors [78]. Therefore, it is likely that RSV uses G to induce let-7f to inhibit the expression of CCND1, DYRK2, ELF4, CCL7 and SOCS3 to favor RSV infection. Although Moore et al. showed SOCS3 protein expression is suppressed by RSV G protein in mouse lung epithelial (MLE)-15 cells [79], while purified RSV G protein induces SOCS3 protein expression in NHBEs in another study [80]. These seemingly contrary findings hint at the complicated multifaceted functions of G in modifying host antiviral responses.

Similar to let-7f, miR-24 also favors RSV replication as the inhibition of miR-24 impairs RSV replication. miR-24 facilitates RSV replication possibly through its complementary binding to the 3′ UTR of DYRK2 directly, leading to the suppression of DYRK2 in A549, and miR-24 could work cooperatively with let-7f to affect DYRK2 expression and viral replication [42]. Furthermore, miR-24 has been considered to directly target IFN-γ in CD4^+^ T cells [81]. It seems that the CX3C motif in G protein was reported to contribute to the induction of let-7f and miR-24 [45]. In polarized Calu-3 cells grown at the air-liquid interface (ALI), recombinant WT RSV induces more let-7 and miR-24 than rA2-GC4, a recombinant RSV containing a point mutation in the CX3C motif at Cys186 (C186S), demonstrating the importance of CX3C motif of G protein in the induction of let-7f and miR-24. Since rA2-GC4 induces less miR-24 than WT, rA2-GC4 is more capable in inducing IFN-γ than WT RSV, further supporting the importance of the CX3C motif of G protein in mediating miR-24-mediated host responses.

Recently, the G protein has also been shown to regulate the induction of miR-26b by RSV. The expression of miR-26b in WT-infected PBMCs is significantly higher than that in cells infected with RSV-ΔG [50]. PMBC samples from RSV children exhibit more miR-26b and less TLR4 than those from healthy children, and the miR-26b level is negatively correlated with the TLR4 mRNA level (R^2^ = 0.5169). Luciferase-UTR assays confirmed that TLR4 is targeted by miR-26b in PBMCs and the TLR4 mRNA level is higher in RSV-ΔG-infected cells compared with WT. All these results demonstrate that the G protein induces miR-26b to suppress TLR-4 as one of the mechanisms to favor RSV infection. Furthermore, the induction of CCL5 and IFN-β, two key downstream products of TLR4, is significantly enhanced by miR-26b inhibitor in RSV-infected cells and RSV-ΔG induces more CCL5 and IFN-β than WT. In clinical samples, the negative correlation between miR-26b and plasma IFN-β (*R*^2^ = 0.4777) and CCL5 (*R*^2^ = 0.5023) concentrations were also observed, supporting that the G protein likely decreases the induction of CCL5 and IFN-β by inducing miR-26b to target TLR-4-mediated signaling.

The importance of NS1/NS2 in miRNA induction/function. Other than RSV G protein, RSV NS1 and NS2 proteins are also involved in regulating host miRNA expression. Both NS1 and NS2 impair let-7i and miR-30b induction by RSV in NHBEs [40]. Mutant RSV lacking NS1 (RSV-ΔNS1) or NS2 (RSV-ΔNS2) induces more let-7i and miR-30b than WT. Moreover, the induction of let-7i by RSV seems augmented by IFN-β. Combined with previous studies demonstrating the inhibitory roles of NS1 and NS2 in RSV-induced type I IFN signaling [82,83,84], these results suggest that NS1 and NS2 may suppress let-7i induction via inhibition of type I IFN signaling.

NS1 also suppresses miR-24 expression to regulate the expression of its target Kruppel like factor 6 (KLF6). RSV-ΔNS1 induces more miR-24 and less KLF6 in A549 than WT, while the NS1 overexpression suppresses the miR-24 expression [43]. The interaction between miR-24 and its target KLF6 seems bidirectional [43], as silencing KLF6 by siRNA significantly increases miR-24 and decreases its downstream effector TGF-β and viral replication, suggesting that NS1 induces KLF6 to suppress miR-24 expression and enhance TGF-β. Interestingly, TGF-β1 stimulation leads to the induction of both KLF6 and miR-24, suggesting the complicated interplay among RSV, miR-24, KLF6, TGF-β, and also other possible unidentified molecules in the network.

RSV NS1 also regulates miR-29a [44]. RSV infection significantly increases miR-29a expression in A549, but RSV-ΔNS1 fails to do so, suggesting that NS1 is responsible for the miR-29a induction. Induced miR-29 seems to target IFNAR1, a key molecule serving as one of two chains of a receptor for IFN-α/β to mediate the type I IFN signaling, because the overexpression of NS1 impairs IFNAR1 expression while the miR-29a inhibitor abolishes NS1-mediated IFNAR1 downregulation. miR-29a suppression also leads to attenuated RSV replication. All these results support that RSV uses its NS1 to induce miR-29, which targets IFNAR1 to enhance RSV replication.

**Figure 1:**
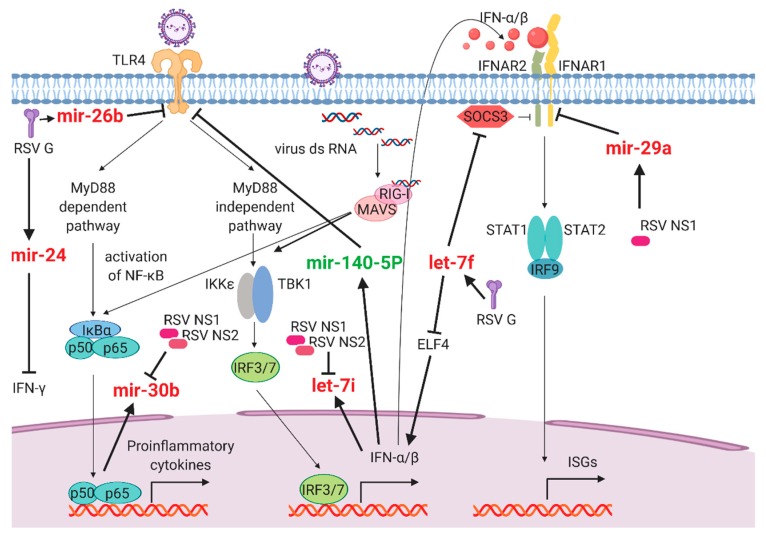
Schematic diagram of the respiratory syncytial virus (RSV)-mediated microRNAs (miRNAs)-messenger RNA (mRNA) interaction networks involved in inflammatory responses. The RSV-increased miRNAs are indicated in red, the decreased miRNAs by RSV are shown in green. Arrows mark the positive effects between elements, whereas stop bars denote inhibitory effects.

**Figure 2:**
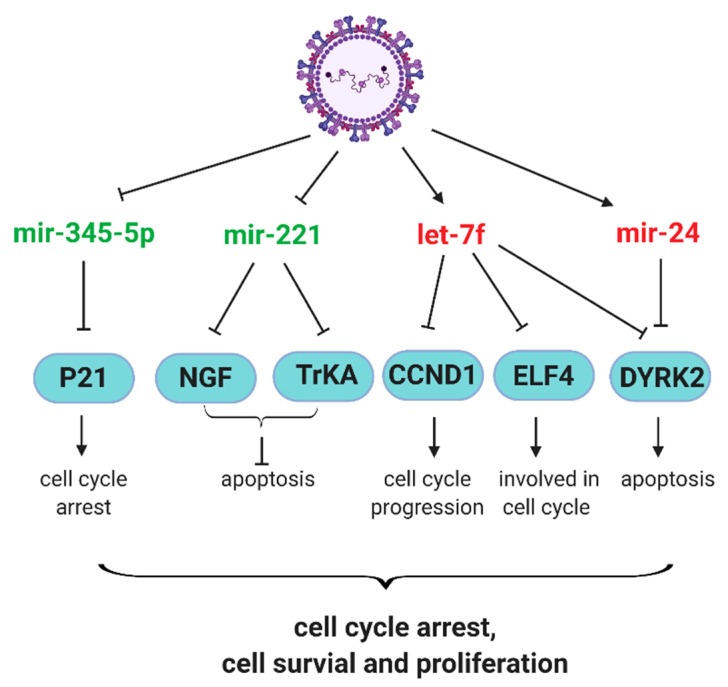
Schematic diagram of the effect of RSV-regulated miRNAs on cell survival. RSV-induced and -decreased miRNAs are indicated in red and green, respectively. Arrows mark and stop bars respectively denote the positive and negative effects on the downstream molecules.

In summary, all studies described above have demonstrated that many RSV- and hMPV-regulated miRNAs are critical for modifying host responses. In Figure 1 and Figure 2, some affected cellular signaling and survival-related paths are respectively summarized, together with the information on how viral proteins contribute to miRNA-mediated pathways.

## 3. tRFs and Their Roles in RSV Infection

**Figure 3:**
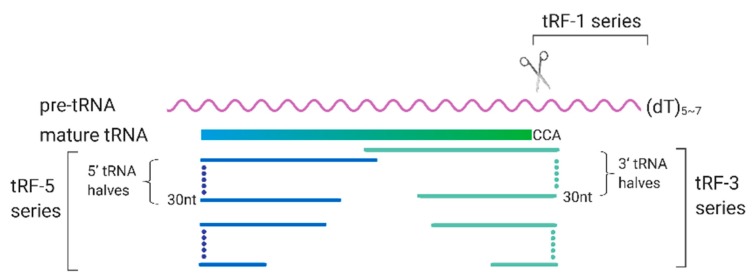
Biogenesis and classification of transfer RNA (tRNA)-derived RNA fragments (tRFs). The tRFs are generally classified into tRF-1 series, tRF-3 series, and tRF-5 series. tRF-1 series are usually those from the 3′-trailer sequences of pre-tRNA. tRF-3 and tRF-5 series are those whose sequences aligned to the 3′- and 5′- end of the mature tRNAs respectively; The length of tRFs ranges from 18 to 40 nt.

tRFs are an emerging family of sncRNAs that are critically involved in many biological processes. They derive from either pre-tRNA or mature tRNAs. As shown in Figure 3, the tRFs are generally classified into tRF-1 series, tRF-3 series, and tRF-5 series [85].

tRFs exhibits multiple biological roles with underlying molecular mechanisms largely unknown. A subset of tRFs has been shown to have a gene *trans*-silencing function using AGOs [86]. Since AGOs are a common platform to some miRNAs and tRFs, tRFs have been reported to affect miRNA-mediated gene silencing by competitively binding to Ago proteins [87]. tRFs also have been found to suppress the stability of multiple mRNA transcripts in breast cancer cells by displacing the 3’ untranslated regions (UTRs) of targets from the RNA-binding protein called Y box binding protein 1 (YBX1) [88]. Besides the effect on mRNAs stability, tRFs also participate in the modulation of translation initiation and elongation [89,90,91,92], and function like piRNAs to interact with Piwi proteins and emerge as a novel apoptosis suppressor [93,94].

RSV infection leads to considerable changes in sncRNA profiles in A549 [95]. In mock cells, the majority of sncRNAs belong to miRNAs (65.4% of total reads), tRFs only account for 1.5% of total reads. In response to RSV infection, tRFs become the most abundant type of sncRNAs (34.1% of total reads) and miRNAs are only 6.4%. Among increased tRFs, most of them derived from 5′-end of mature tRNAs, i.e., tRF5s [95]. Three tRFs derived from the 5′end of tRNAs GluCTC, GlyGCC, and LysCTT (namely, tRF5′-GluCTC, tRF5-GlyGCC, and tRF5-LysCTT, respectively) have been shown to have a gene *trans*-silencing function like miRNAs, but with different regulatory mechanisms. It is commonly known that the 5′-portion of miRNAs are generally important in gene suppression. Unlike miRNAs, the 5′- portion of tRFs seems not so important [95,96]. Another significant difference between tRFs and miRNAs is their biogenesis mechanism. miRNAs production is known to be regulated by Drosha/Dicer dominantly, while the induction of tRF5-GluCTC, -GlyGCC, and -LysCTT by RSV is dependent on a ribonuclease called angiogenin (ANG) [95,96]. It is also interesting that the effect of ANG on tRNA cleavage is tRNA structure-dependent as the cleavage happens often in the anticodon loop of tRNAs, producing about 30 nt-long tRFs. In contrast to RSV, hMPV does not induce these tRF5s [95,96], suggesting the induction of tRFs is virus-specific.

**Figure 4:**
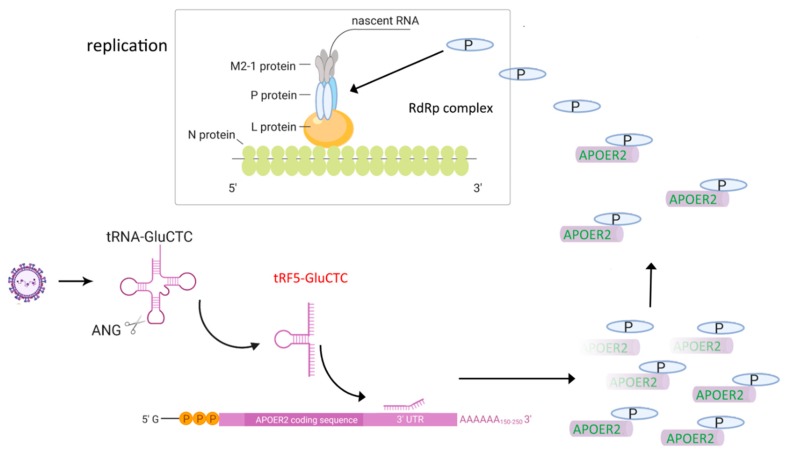
Model on the molecular mechanism used by tRF5-GluCTC to promote RSV replication. RSV infection induces tRF5-GluCTC, which targets APOER2 and suppresses its expression. APOER2 is an antiviral protein carrying out its antiviral role via its sequestration of the P protein of RSV. Therefore, the decreased expression of APOER2 frees more P proteins and makes them more available to form RdRp with other viral proteins.

The induction of tRFs is also replication-dependent, as UV-inactivated RSV fails to induce tRFs. Northern blot assays of RNA samples from the nuclear and cytosolic fractions demonstrated that RSV-induced tRFs exist exclusively in the cytoplasm [95]. An antisense oligonucleotide against tRF5-GluCTC leads to decreased RSV yield and suppressed induction of IL-8, RANTES and IFN-β [95,97], confirming the biological roles of tRF5-GluCTC in RSV infection. By sequencing tRF5-GluCTC-associated RNAs, several potential targets of tRF5-GluCTC are identified. Since tRF5-GluCTC has a gene *trans*-silencing function, previous RSV microarray data was incorporated into the analysis, generating a new set of targets that are commonly present in tRF5-GluCTC-complex and RSV-downregulated mRNA dataset. These targets were then ranked in favor of the interaction energy between the target and tRF5-GluCTC and apolipoprotein E receptor 2 (APOER2) was chosen for the verification [97]. The assay using a luciferase reporter containing the sequence complementary to the predicted target region of APOER2 revealed the luciferase expression to be sensitive to tRF5-GluCTC. The mutagenesis study confirmed the targeting specificity of tRF5-GluCTC. To assess the consequence of the interaction between APOER2 and tRF5-GluCTC, the function of APOER2 was investigated and APOER2 was discovered to interact with RSV P protein, leading to the sequestration of the P protein away from the formation of the RNA-dependent RNA polymerase (RdRp) complex and subsequent suppression of RSV replication. In summary, RSV uses induced tRF5-GluCTC to suppress the APOER2 expression, allowing more P proteins available for the RdRp formation to promote RSV replication (Figure 4) [97]. Similar to tRF5-GluCTC, both tRF5-GlyGCC and tRF5-LysCTT also favor the RSV replication and contribute to RSV-induced inflammation. Their induction is also ANG-dependent and exclusively happens in the cytoplasmic compartment [96].

Other than these three tRFs, our recent unpublished data also confirm the role of tRF5-GlnCTG in RSV replication. Surprisingly, its induction was not ANG-dependent but relied on ELAC2. The mechanism by which ELAC2 uses to generate tRF5-GlnCTG is currently under the investigation.

## 4. Other ncRNAs in RSV Infection

### 4.1. RSV Alters Exosomal piRNAs

piRNAs are single-stranded sncRNAs of 24–32 nts. They interact with Piwi proteins, which belong to the Argonaute/Piwi family, to form the piRISC complex [98]. piRNAs are involved in the silencing of retrotransposons, both at the post-transcriptional and epigenetic levels [99]. In addition, piRNAs also have been shown to regulate genes via DNA methylation modification and mRNA deadenylation [100,101]. Besides miRNAs, the piRNAs in AEC-derived exosomes are also significantly affected by RSV infection [55]. Among exosomal sncRNAs, there are 3.9% and 34.7% piRNAs respectively in control and RSV exosomes. The next-generation sequencing revealed that 52 piRNAs (>10 reads) are commonly present in both mock and RSV exosomes, with 28 upregulated and 24 downregulated piRNAs in RSV exosomes, among which, piR-32678 and piR-59169 show the greatest increase and decrease in expression respectively. Moreover, there are 3 and 19 piRNAs that are uniquely present in mock and RSV exosomes, respectively. Whether and how these affected piRNAs contribute to RSV disease are currently unknown.

### 4.2. Virus-Encoded sncRNAs

Several viruses, the majority of which are DNA viruses, produce virus-encoded sncRNAs to promote their replication or latency [102]. It is commonly believed that negative-sense RNA viruses do not produce viral sncRNAs, because their replication does not occur in the nuclear compartment. To date, there is no report for the existence of RSV-encoded sncRNAs. However, our ultrahigh-throughput sequencing data revealed that hMPV produces several hMPV-encoded sncRNAs. The induction of two hMPV-encoded sncRNAs, respectively derived from the P and L gene, was confirmed by northern blot assay [66]; respectively derived from the P and L gene, were confirmed by northern blot assay [66]. The transcription and replication of hMPV usually occur in the cytoplasm. Therefore, nuclear RNases, such as Drosha and RNase P, cannot access to viral RNAs. Cytoplasmic RNase(s) may be involved in the biogenesis of hMPV-derived sncRNAs. Recently, our group found that two cytoplasmic RNases exoribonuclease 1 (XRN1) and Dicer play a role in generating hMPV-encoded sncRNAs [66,103]. However, whether these hMPV-encoded sncRNAs are functional is not known, as the functions of hMPV-encoded sncRNAs are hard to be experimentally defined. A general method to study the function of an interested ncRNA is to use the antisense oligonucleotides to change its expression or activity, followed by function assays. As the antisense oligonucleotides against viral-derived sncRNAs also can interact with viral genomes, potentially leading to changes in hMPV replication, the visible effects of antisense oligonucleotide treatment do not necessarily result from inhibiting hMPV-derived sncRNAs. Another way to examine the function of hMPV-encoded sncRNAs is to eliminate their induction by mutating the corresponding sncRNA region in hMPV; however, given the importance of L and P in hMPV viral RNA synthesis, it is highly possible that the recombinant mutants cannot be recovered.

### 4.3. lncRNA Involved in RSV Infection

lncRNAs, whose length spans from 200 nt up to 100 kilobases, also emerged as gene regulators, exhibiting multiple regulatory functions on chromatin-remodeling, epigenetic modification, RNA transcription and processing, mRNA stability and translation, protein localization, and miRNAs function [104]. Recently, a study, using NPAs samples from 104 RSV bronchiolitis patients (aged 6.5 ± 4.1) and 40 healthy controls (aged 6.1 ± 3.8), revealed that RSV samples have less maternally expressed gene 3 (MEG3), a broadly studied lncRNA, and more TLR4 than control group samples [105]. Furthermore, it seems that the lncRNA MEG3 suppresses TLR4 expression and plays a partial role in activating NF-κB and MAPK signaling, suggesting the nasal cells use MEG3 to enhance TLR4 expression to carry out antiviral or immune responses. [105].

## 5. Can ncRNAs Serve as Targets for Antiviral Therapeutics?

Conserved viral proteins often serve as antiviral therapeutic targets. Alternative antiviral drug development is host molecule-based. For example, several groups reported that drugs targeting p38 MAPK (mitogen-activated protein kinase), such as berberine, NJK14047, and *N*-acetyl-l-cysteine (NAC), significantly inhibit many respiratory viral infections including RSV replication [106,107,108,109].

Since many ncRNAs also control RSV replication and associated host responses, targeting these ncRNAs can potentially serve as a therapeutic approach [110]. Right after the discovery of ncRNAs, many ncRNAs have been tested for their efficiency in antiviral control. Meanwhile, ncRNA-based drug development was also carried out [111]. Some ncRNA-based drug development showed promising antiviral effects. For example, miRavirsen, an experimental drug targeting miR-122 for HCV infection, was developed and tested in Phase II clinical trials right after the discovery of the effect of miR-122 on HCV replication [112]. The mimics of miR-124, miR-24, and miR-744 also exhibit notable broad-spectrum antiviral activity against several respiratory virus, including influenza A/WSN/1933 (WSN) H1N1, A/Puerto Rico/8/1934 (PR8) and A/Udorn/307/1972 (Udorn) H3N2, RSV-A2, and RSV BT2a, with unidentified underlying mechanism [113]. Whether targeting functional ncRNAs could be a promising anti-RSV strategy that needs to be further experimentally validated.

## 6. Conclusions

RSV and hMPV closely resemble each other, and the clinical manifestations of these two viruses are often indistinguishable. However, these two viruses have unique impacts on host ncRNAs expression. RSV induces tRFs expression, but no viral sncRNAs are detectable by high-throughput RNA sequencing. On the contrary, hMPV fails to induce tRFs, but produces viral sncRNAs. These two viruses both induce let-7f expression. However, the effect of let-7f on RSV and hMPV replication are opposite. These findings suggest that different infection strategies are used by these two respiratory viruses. The studies in clinical samples reveal the potential of miRNAs to be used as diagnostic markers. However, the overall knowledge of the functions of ncRNAs in RSV/hMPV infection is very limited. For example, the molecular mechanisms underlying ncRNAs-associated host responses are still largely unknown. The interaction among ncRNAs, RSV/hMPV, and other host factors are also poorly understood, although the related information is important for target identification and the development of antiviral therapeutics.

## Supplementary Materials

The following are available online at https://www.mdpi.com/1999-4915/12/3/345/s1.
